# Interactions between Cytokines, Congenital Anomalies of Kidney and Urinary Tract and Chronic Kidney Disease

**DOI:** 10.1155/2013/597920

**Published:** 2013-08-26

**Authors:** Ana Cristina Simões e Silva, Flávia Cordeiro Valério, Mariana Affonso Vasconcelos, Débora Marques Miranda, Eduardo Araújo Oliveira

**Affiliations:** ^1^Pediatric Nephrology Unit, Department of Pediatrics, Federal University of Minas Gerais (UFMG), 30130-100 Belo Horizonte, MG, Brazil; ^2^National Institute of Science and Technology of Molecular Medicine (INCT-MM), Faculty of Medicine, UFMG, 30130-100 Belo Horizonte, MG, Brazil; ^3^Interdisciplinary Laboratory of Medical Investigation, Avenida Alfredo Balena, 190, 2nd Foor, Room No. 281, Faculty of Medicine, UFMG, 30130-100 Belo Horizonte, MG, Brazil

## Abstract

Fetal hydronephrosis is the most common anomaly detected on antenatal ultrasound, affecting 1–5% of pregnancies. Postnatal investigation has the major aim in detecting infants with severe urinary tract obstruction and clinically significant urinary tract anomalies among the heterogeneous universe of patients. Congenital uropathies are frequent causes of pediatric chronic kidney disease (CKD). Imaging techniques clearly contribute to this purpose; however, sometimes, these exams are invasive, very expensive, and not sufficient to precisely define the best approach as well as the prognosis. Recently, biomarkers have become a focus of clinical research as potentially useful diagnostic tools in pediatric urological diseases. In this regard, recent studies suggest a role for cytokines and chemokines in the pathophysiology of CAKUT and for the progression to CKD. Some authors proposed that the evaluation of these inflammatory mediators might help the management of postnatal uropathies and the detection of patients with high risk to developed chronic kidney disease. Therefore, the aim of this paper is to revise general aspects of cytokines and the link between cytokines, CAKUT, and CKD by including experimental and clinical evidence.

## 1. Introduction

Fetal hydronephrosis is the most common anomaly detected on antenatal ultrasound, affecting 1–5% of pregnancies [1, 2]. Despite their high frequency of occurrence, there is little consensus on the management of infants with prenatal hydronephrosis (PNH) [3]. There have been a number of studies discussing the significance of fetal renal pelvic dilatation (RPD) as an indicator of urinary tract anomalies [4–7]. The degree of PNH varies from mild to severe, and intuitively, the degree of PNH should correlate with the severity of the underlying etiology [1, 2, 8]. More specifically, the risk of ureteropelvic junction obstruction (UPJO) increased significantly with greater degrees of PNH [9], but the risk of vesicoureteral reflux (VUR) was not significantly different among all severity groups. Most studies also have shown that a single postnatal US is unable to predict the presence or severity of VUR [6, 10, 11]. Consequently, postnatal management is heterogeneous, with some centers advocating detailed investigations including voiding cystourethrography (VCUG) in all cases and others indicating a less intensive approach [12–16]. Therefore, in spite of advances, the issue of postnatal diagnostic management of antenatal hydronephrosis remains a challenging problem [17, 18]. 

RPD can be an early sonographic sign of urinary tract obstruction or as a marker of other abnormalities such as renal duplication or VUR, which cannot be easily identified by US during pregnancy. Therefore, the patient is now presenting to the urologist or pediatric nephrologist before the baby is even born, with a presumptive diagnosis rather than a symptom [19]. Consequently, infants diagnosed with PNH routinely undergo postnatal imaging evaluation. Classically, the prenatal diagnosis of hydronephrosis leads to postnatal investigations, including sonography, VCUG, and isotopic renography [17, 20]. Postnatal investigation has the major aim at detecting infants with severe urinary tract obstruction and clinically significant congenital anomalies of the kidney and urinary tract (CAKUT) among the heterogeneous universe of patients. Imaging techniques clearly contribute to this purpose. However, some of these exams are invasive and very expensive. Furthermore, sometimes imaging techniques are not sufficient to precisely define the indication of surgical approach as well as to determine the prognosis [21].

 Biomarkers have recently become a focus of clinical research as potentially useful diagnostic tools in pediatric urological diseases [22]. Biomarkers are any tests that help distinguishing between two or more biological states and guide further clinical decision making [23]. In this regard, Muller et al. have reported that fetal serum ss2-microglobulin and cystatin C are good markers for postnatal renal function in bilateral renal hypoplasia and dysplasia [24]. More recently, Mersobian et al. [25] searched for specific proteins altered in UPJO by urinary proteome analysis and found a statistically significant difference in the expression of a number of urinary proteins and polypeptides between patients with UPJO and controls. These differences persisted at presentation and through time, although the profile of the candidate biomarkers varied according to the age of the patient. Further studies are needed to identify, among this group of proteins and polypeptides, which potential biomarker can help clinical decisions [25]. For instance, preliminary investigations looking at the urinary concentrations of transforming growth factor-beta (TGF-*β*) have suggested that this cytokine might be useful in detecting urinary tract obstruction and clinically relevant urinary tract anomalies among the heterogeneous universe of patients [26]. 

The obstructive nephropathy is not a simple result of mechanical impairment to urine flow but a complex syndrome resulting in alterations of both glomerular hemodynamics and tubular function caused by the interaction of a variety of vasoactive factors and cytokines that are activated in response to obstruction. The cytokines play a role in the development and progression of fibrotic and sclerotic changes in the obstructed kidney [27]. A large number of factors can initiate apoptosis, several of which may be related to obstructive nephropathy, such as hypoxia, ischemia, cytokines, growth factors, angiotensin II, endothelin-1, thromboxane, prostaglandins, and mechanical stretch [28–30]. However, it should be pointed that the biochemical, cellular, and molecular mechanisms of the obstructive uropathies are still largely unknown [28, 31]. The understanding of this process will certainly help in the management of fetal hydronephrosis and in the detection of patients at high risk for chronic kidney disease (CKD). In this regard, recent studies suggest a role for cytokines and chemokines in the pathophysiology of fetal hydronephrosis [28, 31, 32]. Indeed, we believe that the evaluation of these inflammatory mediators might help the management of CAKUT. The aim of this paper is to revise general aspects of cytokines and the link between cytokines, CAKUT, and CKD by including experimental and clinical studies. For this purpose, we have searched for articles at PubMed and Scopus by using the combination of words: “UPJO,” “VUR,” or “CAKUT” and “chemokines” or “cytokines.” After this first step, we have selected the papers that evaluated cytokines as potential markers of clinical course, urinary tract obstruction, and/or CKD in pediatric patients. In that way, we have composed the list of papers presented in this review. 

## 2. Cytokines: General Concepts and Characteristics

Cytokines are redundant secreted proteins with growth, differentiation, and activation functions that regulate and determine the nature of immune responses and control the immune cell trafficking and the cellular arrangement of immune organs. These mediators are involved in virtually every facet of immunity and inflammation, including innate immunity, antigen presentation, bone marrow differentiation, cellular recruitment and activation, and adhesion molecule expression. A cascade of responses is triggered in response to cytokines, and several cytokines acting together are required to express their optimal function. Numerous cytokines have proinflammatory properties such as interleukin-6 (IL-6) and tumor necrosis factor alpha (TNF-*α*), whereas others modulate the inflammatory response-like Interleukin-10 (IL-10) and transforming growth factor beta (TGF-*β*) [33]. 

Chemokines constitute a large family of low molecular-weight cytokines whose main action is the recruitment and activation of leukocyte subsets in various models of inflammation—the word “chemokine” is a contraction of the terms “chemoattractant” and “cytokine” [34]. Tubular epithelial cells can be a rich source of inflammatory chemokines including regulated on activation, normal T expressed and secreted (CCL5/RANTES), monocyte chemotactic protein-1 (CCL2/MCP-1), Macrophage inflammatory protein 1 alfa (CCL3/MIP-1*α*), CX3CL1/fractalkine, and interleukin-8 (CKCL8/IL-8) [35]. Tubular epithelial cells are also targets for chemokines, since these cells respond to CCL2/MCP1 stimulation by releasing IL-6 and intracellular adhesion molecule-1 [36]. Messenger RNA for chemokine receptors can also be detected in podocytes and glomeruli [34].

## 3. Cytokines in Renal Diseases Related to CAKUT

A number of studies have shown the relation between renal diseases and cytokines production [28, 34, 37–40]. Indeed, the measurement of urinary, plasma, and renal tissue levels of cytokines has been used to monitor and diagnosis various urological and kidney diseases [34, 40, 41]. In this section, we reported studies that associated cytokines with relevant clinical consequences of CAKUT such as acute pyelonephritis, urinary tract obstruction, and renal scarring.

Acute pyelonephritis is most commonly observed in pediatric patients with CAKUT that resulted in urinary tract obstruction. However, predictive factors of renal scarring in patients with acute pyelonephritis remain unknown. In this regard, Sheu et al. [38, 39] evaluated serum and urinary levels of interleukin-1*β* (IL-1*β*), IL-6, and CXCL8/IL-8 in children with acute pyelonephritis. In the first study, these authors reported that the levels of IL1-*β* were significantly reduced in children with renal scarring, probably indicating a protective function for this cytokine [38]. IL-1*β* is primarily synthesized by cells of the mononuclear phagocyte lineage, but endothelial cells and neutrophils also produce this cytokine. The most important biological activity is its ability to activate T lymphocytes and to augment B-cell proliferation thus increasing immunoglobulin synthesis [33]. These effects might be responsible for a protection against renal scarring in patients with acute pyelonephritis. On the other hand, the same research group previously found that there is a significant elevation of serum and urinary levels of IL-6 and CXCL8/IL-8 in children with acute pyelonephritis when compared to children with lower urinary tract infection [39]. This finding supports the hypothesis that the release of IL-6 and of CXCL8/IL-8 from the urinary tract leads to systemic host responses [39], since IL-6 is a proinflammatory cytokine responsible for pyrexia and production of acute phase proteins [33], whereas CXCL8/IL-8 is a chemokine responsible for neutrophil infiltration into the urinary tract with an important role in acute inflammation [39]. In addition, gene polymorphisms of CXCL8/IL-8 seem to increase the susceptibility for acute pyelonephritis. For instance, the presence of the IL-8-251A allele in the genotype of children with urinary tract infection without vesicoureteral reflux has increased the risk of pyelonephritis [42].

In relation to renal scarring, TGF-*β* is a fibrogenic cytokine that stimulates extracellular matrix proteins deposition and scarring formation in kidney parenchyma. On the other hand, concerning immune system regulation, TGF-*β* exerts anti-inflammatory effects by inhibiting the proliferation of many different cell types [33]. Besides renal scarring, TGF-*β* also seems to be related to urinary tract obstruction. In this regard, Monga et al. [43] have studied 17 men with bladder outlet obstruction and 6 nonobstructed subjects and showed that, in the obstructed ones, the urinary levels of TGF- *β* were significantly higher than in non-obstructed. 

## 4. Cytokines in CAKUT: Experimental Studies

 Animal models have been frequently used to understand histopathological changes, mechanisms, and therapeutic approaches of obstructive nephropathies [41, 44–46]. The majority of the reported animal models utilized rats and mice, but rabbits, pigs, and sheep were also used [31]. 

 Models of experimental postnatal unilateral ureteral obstruction have been developed in newborn rat pups that continue to exhibit active nephrogenesis in the postnatal period [31]. A partial unilateral ureteral obstruction was surgically created by entrapping the ureter in the animal psoas muscle, whereas the complete obstruction was produced by surgically clamping and occluding the ureter [31]. In rats, the major part of nephrogenesis occurs within 7 to 10 days after birth [47, 48]. Some models have used animals with congenital uropathies, while others have evaluated animals submitted to surgery after birth [47].

**Figure 1:**
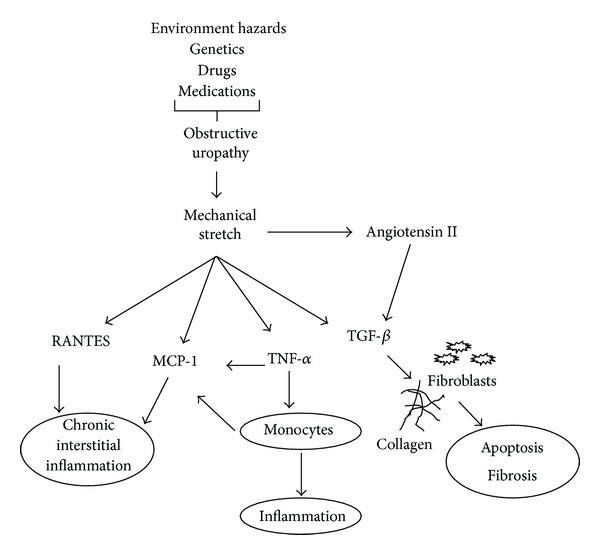
Potential mechanisms involved in obstructive uropathies.

 The induction of ureteral obstruction in newborn rats clearly interferes with ongoing nephrogenesis and this procedure usually leads to substantial renal damage [47]. This kind of experimental model mimics human ureteral obstruction at the second and third trimesters of pregnancy; however, significant renal damage is less common in infants [49]. The main features found in obstructive models are tubular cell apoptosis, mesenchymal myocyte transformation, and decreased glomerular endowment and glomerular injury [28, 48, 50]. The understanding of the pathophysiological mechanisms and the molecular events is important to define the moment of intervention [31].  Figure 1 shows the main mechanisms involved in models of obstructive uropathies.

Obstructed kidneys exhibited an elevation in angiotensin II activity, which, in turn, decreases renal blood and causes ischemia and kidney growth arrest. Although renal blood flow usually normalizes 6 weeks after the relief of temporary obstruction, renal growth remains altered, suggesting that other factors are responsible for growth impairment [47] such as the reduction in cell proliferation, the increase in cell apoptosis, and the progression of interstitial fibrosis [48].

 Chevalier et al. [48] have studied neonatal rats submitted to unilateral ureteral obstruction or sham operation at one day of age, with relief five days later. In additional groups of neonatal rats, the operation was at 14 days, with relief at 19 days [48]. Three months following relief of unilateral ureteral obstruction during days 14 to 19, renal growth was decreased by 50%, compared to a 30% reduction following relief of unilateral ureteral obstruction during days 1 to 5. The number of glomeruli was reduced by approximately 50% regardless of the timing of obstruction, but glomerular size was reduced only in rats with unilateral ureteral obstruction from days 14 to 19 [48]. This study shows that, in the period immediately following nephrogenesis, the kidney is particularly susceptible to long-term injury from temporary unilateral obstruction. This suggests that a delay in relief of significant ureteral obstruction should be avoided if diagnosed in the perinatal or neonatal period [48]. The same group has also evaluated neonatal rats that underwent unilateral ureteral obstruction at one day of age whose obstruction was released at days 1, 2, 3, or 5 following the operation [51]. The growth of the obstructed kidney decreased linearly according to the duration of ureteral obstruction, while the contralateral kidney developed compensatory hypertrophy [51]. Indeed, contralateral renal hypertrophy should be considered as an important sign of advanced obstructive uropathy [52]. In summary, these animal models reveal that renal growth and function are impaired in proportion to the severity and duration of obstruction.

 The microscopic alterations of obstructed kidneys are, initially, increased of tubular diameter secondary to tubular cell proliferation and dilatation. Next begins the apoptosis of tubular cell followed by the apoptosis of interstitial compartment [53]. There is a gradual, but continuous, apoptosis and proliferation of fibroblasts and inflammatory cells [53, 54]. Tubular cell apoptosis contributes to renal growth impairment [53], whereas proliferation of interstitial fibroblasts with myofibroblast transformation leads to excess deposition of the extracellular matrix and renal fibrosis [50]. Phenotypic transition of resident renal tubular cells, endothelial cells, and pericytes has also been implicated in this process. 

A variety of intrarenal factors lead to progressive interstitial fibrosis, including growth factors and cytokines, such as angiotensin II, MCP-1, TGF-*β*, and adhesion molecules, which are produced by the hydronephrotic kidney [28]. Altered renal expression of growth factors and cytokines modulate cell death by apoptosis or phenotypic transition of glomerular, tubular, and vascular cells. Mediators of cellular injury include hypoxia, ischemia, and reactive oxygen species, while fibroblasts undergo myofibroblast transformation with increased deposition of extracellular matrix. On the other hand, a number of endogenous antifibrotic counter-regulatory molecules have been identified, opening the possibility of enhancing the kidney's own defenses against progressive fibrosis [28, 55].

Cytokines as TGF-*β* and TNF-*α* and chemokines like CCL2/MCP-1, CCL5/RANTES, macrophage inflammatory protein-2 (MIP-2), and *γ*-interferon-inducible protein (IP-10) have been evaluated in experimental hydronephrosis [27, 28, 31, 32].

TGF-*β* is highly involved in tubulointerstitial fibrosis. This cytokine increases matrix synthesis, collagen deposition, and tubular apoptosis, upregulates the integrin-matrix adhesion, and inhibits matrix degradation [32, 45, 56]. Resident renal tubular cells and interstitial cells may be responsible for TGF-*β* production; however, interstitial fibroblast cells seem to be the major source of TGF-*β* during the process of interstitial fibrosis [57]. In this regard, Mizuno et al. [58] found that the increased expression of TGF-*β* was correlated to fibrotic changes of interstitial regions in kidneys of mice subjected to unilateral ureteral obstruction. Accordingly, Seseke et al. [50] also detected the association between interstitial fibrosis and increased renal expression of TGF-*β* mRNA in an inbred strain of rats with congenital hydronephrosis. In addition, Zhou et al. [52] reported a marked elevation of renal TGF-*β* level in parallel to fibrotic changes of congenital and surgical ureteral obstruction in rats. Indeed, TGF-*β* expression increased significantly after completing nephrogenesis [47].

The role of TGF-*β* in obstructive nephropathies was also evidenced in other animal species. Seremetis and Maizels [56] have studied rabbit pups submitted to left partial ureteral constriction and human specimens of renal pelvis and ureter derived from cases of isolated renal obstruction managed by pyeloplasty and nephrectomy or of isolated vesicoureteral reflux managed by ureteral reimplantation. These authors have detected significantly higher expression of TGF-*β* mRNA in obstructed pelvis than in nonobstructed ones. This elevation in TGF-*β* mRNA expression was correlated to muscle hypertrophy and increased collagen deposition, both representing the process of renal pelvis remodeling in response to obstruction. The lower level of TGF-*β* mRNA expression may be a sign of less remodeling due to a steady state of obstruction. The expression of TGF-*β* mRNA emerges as a good predictor of early obstruction [56]. 

The molecular pathways for TGF-*β* receptor-mediated effects were also evaluated in experimental hydronephrosis [31]. In this context, Smad 3 is a protein responsible for signaling downstream of the TGF-*β* receptors [59]. Sato et al. [60] have studied mice with genetic deletion of Smad3 and the wild type controls. The right proximal ureter was exposed and double ligated at 6–8 weeks of age. In the absence of Smad3, the formation of fibroblasts was blocked, clearly indicating a connection between fibrosis and TGF-*β* in obstructive uropathies [60]. In our point of view, animal models of CAKUT support the role of TGF-*β* as a potential biomarker for urinary tract obstruction. We also believe that translational studies should be done in order to establish the role of TGF-*β* in human CAKUT pathogenesis and to search for alternative pharmacological targets by inhibiting this cytokine. 

TNF-*α* may play a role in initiating tubulointerstitial injury in obstructed kidney [28]. TNF-*α* stimulates the production of chemotactic factors by resident cells and upregulates CCL2/MCP-1 in human mesangial cells [28]. The increase of TNF-*α* at early stages of obstruction stimulates the production of chemoattractants for monocytes, which in turn contributes to leukocyte infiltration in obstructed kidneys [28]. Misseri et al. [61] have studied the expression of TNF-*α* mRNA in rats submitted to progressive degrees of left ureteral obstruction. Renal cortical TNF-*α* mRNA expression and protein production reached a peak at 3 days of ureteral obstruction. The TNF-*α* production, localized primarily to renal cortical cells, was not associated with significant inflammatory cell infiltrate [61]. Indeed, TNF-*α* might participate in initiating tubulointerstitial injury in the obstructed kidney by upregulating chemoattractants for monocytes and by producing leukocytes infiltration [32]. The data evaluating TNF-*α* are still very limited. However, considering that TNF-*α* has proinflammatory properties, it seems reasonable to investigate the role of this cytokine on the pathways linking tubulointerstitial injuries to CKD.

In relation to chemokines, Vielhauer et al. [62] found an increased expression of the CC chemokines, CCL2/MCP-1, and CCL5/RANTES, at sites of progressive tubulointerstitial damage in murine obstructive nephropathy model. It was also observed an interstitial infiltration of macrophages and T lymphocytes, which differentially expressed the CCR2 receptors. These data suggest that CCR2- and CCR5-positive monocytes and CCR5-positive lymphocytes are attracted by locally released CCL2/MCP-1 and CCL5/RANTES, resulting in chronic interstitial inflammation [62]. Indeed, CCL2/MCP-1 is an inflammatory chemokine that attracts and activates monocytes, T cells, and natural killer cells [33, 34]. In this regard, Stephan et al. [49] produced partial or complete ureteral obstruction in 28-day-old Wistar rats. These authors found that mRNA expression for CCL2/MCP-1 was moderately increased in partial ureteral obstruction, whereas kidneys without significant damage did not show any upregulation [49]. The study qualifies MCP-1 mRNA expression as a prognostic marker of partial ureteral obstruction [49]. On the other hand, Crisman et al. [63] detected the expression of CCL2/MCP-1, CCL5/RANTES, and IP-10 at 1 day of unilateral ureteral obstruction in mice, and, at 7 days, RANTES became the most abundant chemokine in the obstructed kidney [63]. Therefore, more studies still need to clearly define the role of CC chemokines in obstructive uropathies.

Other cytokines had also been associated to experimental models of CAKUT. For example, 75% of transgenic animals with overexpression of IL-9 developed congenital hydronephrosis, and the alteration was dependent on the presence of IL-4 and IL-13 [64]. In addition, Madsen [65] found significantly lower levels of IL-10 in renal parenchyma and urine of acute unilateral obstructed animals, while renal levels of IL-1*β*, IL-6, and TNF-*α* were increased to sham-operated animals. 

The study of cytokines in hydronephrosis might provide new insights for the treatment or novel ways to blunt renal damage in obstructive uropathies. For instance, animals with right ureter obstruction treated with spironolactona exhibited less fibrosis than control group [46]. Since angiotensin II contributes at least in part to the increased expression of TNF-*α* mRNA in obstructed kidney [28], the use of angiotensin converting enzyme inhibitors emerges as an effective way in preventing renal fibrosis [44]. The use of statins also emerged as potential treatments. In this regard, the administration of atorvastatin ameliorated the tissue damaged of obstructed ureters in an experimental model [66]. The expression of TGF-*β*1 and of the proinflammatory cytokines IL-1*β*, IL-6, and TNF-*α* was decreased following atorvastain treatment [66]. Another rational approach to blunt renal fibrosis is to block growth factors effects. In this regard, Isaka et al. [57] showed that interstitial fibrosis could be blocked by TGF-*β*1 antisense oligodeoxynucleotides. 

Additionally, the modulation of nitric oxide, epidermal growth factor (EGF), and hepatocyte growth factor seems to be a good strategy to treat obstructive nephropathy in the future [55, 58, 67]. In summary, there are very few studies on the role of immune markers as therapeutic targets in experimental CAKUT. However, the inhibition of proinflammatory and fibrogenic cytokines seems to be a reasonable strategy to preserve renal function. 

## 5. Cytokines in CAKUT: Clinical Studies

 It should be pointed that few data about the role of cytokines in CAKUT were provided by clinical studies and the majority of them evaluated ureteropelvic junction obstruction (UPJO) and vesicoureteral reflux (VUR).

### 5.1. Ureteropelvic Junction Obstruction

 UPJO is the most common cause of severe hydronephrosis in children [68]. UPJO is unilateral in 90% of cases and may result from intrinsic narrowing at the junction between ureter and renal pelvis or extrinsic compression by an accessory lower pole artery of the kidney [21]. The degrees of hydronephrosis vary among patients with UPJO. The histological changes may vary from the absence of abnormalities to renal dysplasia with glomerulosclerosis and extensive interstitial fibrosis and tubular atrophy [69]. The UPJO area is consistently inflamed and has varying degrees of fibrosis and muscular hypertrophy [69].

 Postnatal differentiation between obstructive and non-obstructive hydronephrosis is quite difficult. Several studies have been made in patients with UPJO in order to find out noninvasive biomarkers to allow the diagnosis and treatment of these patients. In this regard, cytokines and growth factors have been studied in UPJO [41]. The most relevant results were obtained with MCP-1, EGF, and TGF-*β*.

 Healthy children presented high expression of EGF mRNA in renal tissue, whereas CCL2/MCP-1 mRNA was normally undetectable. On the other hand, in UPJO patients, CCL2/MCP-1 gene expression was strikingly increased at the tubulointerstitial level, while the EGF gene expression was markedly reduced. The interstitial mononuclear cell infiltrate in UPJO patients was strictly correlated with the degree of tubulointerstitial damage [70, 71]. Accordingly, the urinary concentrations of EGF were reduced in UPJO patients, whereas the CCL2/MCP-1 levels were increased [70, 72]. After surgical correction, there was a significant reduction in urinary levels of CCL2/MCP-1 accompanied by a marked increase in EGF concentration. Therefore, these two cytokines could be useful for the followup of obstructed patients [70]. In a prospective study, Madsen reported that urinary concentrations of EGF and of CCL2/MCP-1 were significantly increased in preoperative samples collected in UPJO patients before surgical procedure in comparison to urine from healthy children [65]. At this same study, the concentrations of CCL2/MCP-1, MIP-1*α*, IP-10, and RANTES were increased in urine from the obstructed kidney compared to urine from the contralateral nonobstructed kidney [65]. These urine samples were collected during the surgical procedure. One year after surgery, the concentrations of EGF, CCL2/MCP-1, MIP-1*α*, IP-10, and CCL5/RANTES were decreased to levels comparable to healthy controls [65, 73]. 

 Taranta-Janusz compared obstructed PNH cases (who underwent surgery) with nonsurgically managed cases and with healthy subjects (control group). These authors found that urinary levels of CCL2/MCP-1 from voided urine before and after surgery and from the affected pelvis were significantly higher than nonsurgically managed cases as well than control group [74]. The authors also studied the level of osteopontin (OPN) and CCL5/RANTES in urine samples. Urinary levels of OPN were significantly higher in surgical cases than in nonsurgically managed patients [74]. Urinary levels of CCL5/RANTES were significantly higher in urine samples from affected pelvis collected during surgery than in voided urine before pyeloplasty [74]. Three months after surgery, the urinary levels of these three biomarkers did not return to control values [74]. 

 Palmer et al. [75] have studied patients who undergoing pyeloplasty (UPJO patients), ureteral reimplantation (VUR patients), or circumcision/orchiopexy and measured urinary levels of TGF-*β*1 collected in bladder and pelvis. TGF-*β*1 concentrations were detected in all groups without significant differences in bladder samples. In contrast, the level of this cytokine was significantly elevated in the renal pelvis of children with UPJO when compared to the level obtained in the bladder of control group, of VUR group, and of UPJO patients [75]. More recently, Furness et al. [76] have measured urinary levels of TGF-*β*1 collected in the bladder and renal pelvis of patients with UPJO. Urinary levels of TGF-*β*1 in children with UPJO were 4-fold higher than in healthy controls, and samples obtained in renal pelvis had a 2-fold increase in cytokine concentrations when compared to bladder samples. In addition, if a cut-off point of 61 pg/mg creatinine was considered, a 92% of sensitivity was obtained for the urinary measurement of TGF-*β*1 in bladder [76]. The main concern of this study was the lack of correlation to patients with dilated nonobstructed uropathy conservatively managed. In a case-control study where 19 patients underwent pyeloplasty, Sager et al. found that when TGF-*β*1 levels were above 39.75 pg/mL, the patients have a 4.25-fold relative risk of having obstructive hydronephrosis compared with levels below 39.75 pg/mL [77]. 

 El-Sherbiny et al. [78] have compared urinary TGF-*β* levels between obstructed and nonobstructed patients with grade 3 hydronephrosis. In obstructed patients, urinary concentrations of TGF-*β* measured in renal pelvis were 4-fold higher than the measurements in the bladder, which were, in turn, 3-fold higher than in healthy controls samples. There was also a trend in decreasing bladder TGF-*β* levels 3 months after surgical correction of obstruction. Furthermore, the measurement of urinary levels of TGF-*β*1 had 80% of sensibility and 82% of specificity for the recognition of obstruction [78]. At the same hospital in Egypt, Taha et al. [79] have evaluated 35 children with UPJO submitted to pyeloplasty who had grade 3 or higher hydronephrosis. These authors have found significantly elevated levels of TGF-*β* in UPJO group compared to healthy controls. The presence of high baseline urinary levels of TGF-*β* in younger children significantly increased the diagnostic accuracy of this measurement. In addition, there was a decrease of TGF-*β* concentration 1 month after of pyeloplasty that reached statistical significance 1 year after surgery [79]. The difference in the results obtained in both Egyptian studies might be due to time-point of the measurements: 3 versus 12 months after pyeloplasty.

 Zieg et al. reported that urinary levels of TGF-*β*1 were significantly higher in patients with obstructive uropathies than in patients with nonobstructive hydronephrosis and healthy controls [80]. A positive correlation between urinary TGF-*β*1 levels and proteinuria was found in obstructive uropathies [80]. 

 Older children normally have lower urinary levels of TGF-*β*1 in the bladder probably due to the reduction or the steady-state production of this cytokine in long-term obstruction [76, 78, 79]. In Canada, Almodhen et al. [26] have evaluated the role of TGF-*β* in the diagnosis and longitudinal followup of a homogeneous group of newborns with prenatal unilateral hydronephrosis. These authors showed that in the conservatively managed group the decrease in hydronephrosis grade through time was associated with a similar decrease in urinary concentrations of TGF-*β*1 [26]. This result indicates the utility of urinary measurement of TGF-*β*1 for monitoring patients with congenital hydronephrosis. In the surgical-treated group, urinary concentrations of TGF-*β*1 significantly decreased after pyeloplasty during a mean followup of 7 months. At a cut-off point of 17 pg/mmol of creatinine, the measurement of urinary TGF-*β*1 in the first 3 months of life had 82% of sensibility and 86% of specificity in predicting surgery [26]. Besides different methodologies and timing of urine collection, TGF-*β*1 is the marker more investigated and promising in discriminating obstructive from nonobstructive CAKUT (Table 1).

### 5.2. Vesicoureteral Reflux

 VUR is a congenital anomaly that increases the risk of repeated pyelonephritis and, consequently, can result in renal scarring, renin-mediated hypertension, and, in some cases, renal insufficiency [81, 82]. VUR is a heterogeneous condition that can be primary or associated with multicystic kidney, hypodysplastic kidneys, renal agenesia, and renal or ureteral ectopia. Kidneys with reflux nephropathy have disjointed glomeruli from proximal tubules, interstitial infiltration with chronic inflammatory cells, and periglomerular fibrosis. Dysplatsic feature is one of the characteristics of congenital reflux nephropathy. The main findings are areas of mesenchymal tissue containing primitive tubules [83].

Associations between gene polymorphisms of TNF-*α*, TGF-*β* and of VEGF with VUR were found [92–96]. Some of these polymorphisms were also associated to reflux nephropathy and progressive renal damage [94, 95]. Hussein et al. showed that specific variants in the promoter regions of the genes encoding TGF*β* (−509T allele) and VEGF (−406CC genotype) were associated with an increased risk for the development of renal scarring [96]. These associations could help in understanding the mechanisms of reflux nephropathy and could allow the detection of patients at risk of CKD.

 TNF-*α* and TGF-*β* are abundant in the smooth muscle cell of the ureter of VUR patients [97]. On the other hand, patients without VUR have higher expression of growth promoting factors like insulin growth factor-1 (IGF-1), nerve growth factor (NGF), and vascular endothelial growth factor (VEGF) than those with VUR [97]. In this regard, Chertin et al. [83] have showed that the reduced production of EGF associated with high expression of CCL2/MCP-1 might cause an overproduction of proinflammatory and profibrotic cytokines that trigger apoptosis, ultimately leading to tubular atrophy and renal dysfunction in reflux nephropathy [83].

 The inflammatory process in VUR is ongoing despite the occurrence or not of urinary tract infection (UTI). The elevated urinary level of CXCL8/IL-8 in children with reflux and without UTI might contribute to reflux nephropathy [84, 87]. Haraoka et al. [84] have found a significant difference between urinary levels of IL-8 in children with and without renal scarring and in patients with and without VUR. Merrikhi et al. [90] also showed significantly higher levels of IL-8 in patients with RVU than in those without RVU. This finding suggests that urinary IL-8 measurements could be useful to detect VUR patients with more pronounced renal damage and who need strict followup [84]. Galanakis et al. [87] proposed the use of IL-8 as a biomarker for the diagnosis of VUR. A cut-off concentration of 5 pg/*μ*mol has a sensitivity of 88% and a specificity of 69% [87]. Our research group has recently reported a correlation between high urinary levels of IL-8/CXCL8 and reduced glomerular filtration rate in CAKUT patients, suggesting that this chemokine might be associated to renal scarring and CKD [98]. 

 IL-6 may also be involved in the pathogenesis of reflux nephropathy. IL-6 induces B and T cells activation and differentiation during inflammation [33]. Ninan et al. [85] have detected a significant elevation of urinary IL-6 levels in patients with VUR. In addition, Wang et al. [86] have found that urinary IL-6 was significantly higher in children with severe bilateral renal scarring than in those with mild scarring and normal controls. Gokce et al. [89] have related high urinary levels of IL-6 with the presence of VUR and increased IL-8 concentrations with renal scarring. Concerning serum measurements of cytokines, Jutley et al. [99] have detected significant elevation of IL-6 and TNF-*α* in patients with reflux nephropathy when compared to those without reflux nephropathy or to healthy controls. 

 Since the main histological alteration in reflux nephropathy is renal fibrosis, Sabasiñska et al. [88] have measured urinary levels of TGF-*β* in patients with VUR. These authors have found that urinary concentrations of TGF-*β* were increased in high-grade reflux and in bilateral cases [88]. Our research group studied the urinary concentrations of TGF-*β*, IL-6, and TNF-*α* in three different groups: idiopathic RPD, urinary tract anomalies, and dysplastic kidneys. TGF-*β* levels tended to be higher in the hypodysplastic kidney group compared to idiopathic RPD, while very similar values for IL-6 and TNF-*α* were found in these groups. On the other hand, urinary levels of TGF-*β* were significantly higher in patients with reduced dimercaptosuccinic acid (DMSA) uptake on technetium-99 m DMSA scintigraphy (AUC 0.67 [95%CI, 0.56–0.79]) [100]. A cut-off value of 2 pg/mL for TGF-*β*1 showed a sensitivity of 82.8% [95% CI, 64.2–94.1] and a specificity of 47.9% [95% CI, 35.9–60.1] for identifying those patients with reduced DMSA uptake [100]. Our findings also support the general idea that TGF-*β* has a role in renal fibrogenic processes.

 Studies about renal scarring and VUR pathogenic process are still scarce making any powerful analysis very difficult. On the other hand, based on the available data, we consider that the proinflammatory cytokines (IL-6 and TNF-*α*), the chemokine, CXCL8/IL-8, and the fibrogenic cytokine, TGF-*β*, should be more intensively evaluated as potential biomarkers for renal scarring and for the emergence of CKD in reflux nephropathy (Table 2).

## 6. Concluding Remarks

 CAKUT accounts for a great fraction of CKD in children [101]. Genetic, inflammatory, fibrogenic, environmental, and epigenetic factors responsible for these lesions are largely unidentified, and attention has been focused on minimizing obstructive renal injury and optimizing long-term outcomes to avoid or, at least, delay the progression of CKD. The renal response to urinary tract obstruction is complex and involves a wide array of interacting molecules in an early timing, being surgical *in utero* interventions performed when renal lesions were already irreversible [102]. 

 New diagnostic approaches to and alternative therapies for CAKUT are clearly necessary. In this context, research into biomarkers has reached great importance. Clinical and experimental lines of evidence leave no doubt about the role of inflammation in renal diseases. Understanding the effects of cytokines on the onset and progression of renal injury is thus paramount, as new prognostic markers and maybe as alternative therapeutic targets.

 Therefore, urine measurements of cytokines seemed to be useful in CAKUT as predictors of urinary tract obstruction and renal scarring. The chemokine CCL2/MCP-1 and the cytokine TGF-*β* have been frequently associated with urinary tract obstruction in patients with UPJO, whereas high urinary levels of IL-6 and of CXCL8/IL-8 were found in many patients with VUR and correlated to renal scarring and to renal function deterioration. 

Yet, in spite of great advances in our knowledge about the pathophysiological mechanism linking the cytokines to CAKUT and CKD, much remains to be elucidated.

## Figures and Tables

**Table 1:** Studies on urinary cytokines in patients with UPJO.

Author	Year	Ref.	Age of patients	Cytokine	Study/control group (*N*)	Sensitivity	Specificity	Conclusions
Palmer et al.	1997	[75]	4.6 years (1 month to 11 years)	TGF-*β*_1_	13/VUR (11) and healthy children (19)	—	—	Pelvic urinary TGF-*β*_1_ levels are elevated compared to the control group

Furness et al.	1999	[76]	Median: 2.1 years	TGF-*β*_1_	30/healthy children (19)	92%	—	Bladder urinary TGF-*β*_1_ levels are significantly elevated

Grandaliano et al.	2000	[70]	1 months to 13 years	MCP-1; EGF	24/healthy children (15)	—	—	Bladder urinary EGF levels are reduced in UPJO, while MCP-1 levels are elevated

El-Sherbiny et al.	2002	[78]	5.2 ± 4.7 years	TGF-*β*_1_	15/dilated non obstructed kidneys (11)	80%	82%	Elevated bladder urinary TGF-*β*_1_ levels in obstructed kidneys decreased after surgery

Taha et al.	2007	[79]	Median: 5.9 years	TGF-*β*_1_; EGF	35/healthy children (30)	100% (TGF-*β*_1_)	80% (TGF-*β*_1_)	Bladder urinary TGF-*β*_1_ levels are significantly elevated, while no significant differences are detected in EGF levels

Almodhen et al.	2009	[26]	14 ± 6 months	TGF-*β*_1_	42/—	82%	86%	Bladder urinary TGF-*β*_1_ levels can predict the need for surgery

Sager et al.	2009	[77]	6.7 years ± 5.6	TGF-*β*_1_	19/no renal pathology (19)	—	—	Bladder urinary TGF-*β*_1_ levels in obstructed patients were higher than in controls, and renal pelvic urinary levels of TGF-*β*_1_ in the hydronephrotic kidney were higher than preoperative bladder urine sample Postoperative TGF-*β*_1_ concentration was significantly lower than preoperative

Bartoli et al.	2011	[72]	Functional UPJO: 55 (34) months; obstructive UPJO: 34 (28) months; underwent pyeloplasty group 80 (52) months; control 31 (23) months	MCP-1; EGF	76/30 healthy	—	—	Obstructive UPJO patients showed increased urinary levels of MCP-1 and decreased urine concentration of EGF. The urine EGF/urine MCP-1 and urine EGF/urine *β*2M ratios were significantly downregulated in untreated UPJO groups compared with control group, as well in the comparison between obstructive versus functional UPJO

Madsen, Madsen et al.	20132012	[65, 73]	8.1 (3.5–15) years at time of surgery	EGF, IP-10, MCP-1, MIP-1*α*, RANTES	28/13 healthy children	—	—	EGF and MCP-1 were significantly increased in preoperative UPJO samples. Concentration of MCP-1, MIP-1*α*, IP-10, and RANTES were increased in obstructed kidney and decreased one year after surgery

Taranta-Janusz et al.	2012	[74]	1.03 (0.08–14) years—surgical cases; 8 (0.75–17) years—conservative cases; 3 (0.33–16) years—control group	MCP-1, OPN, RANTES	15 surgical cases/21 conservative cases/19 control group			Only urinary MCP-1 has good diagnostic accuracy in identifying children with abnormal differential renal function (AUC 0.862) and in detecting kidney injury (AUC 0.704). MCP-1 levels from voided urine before and after surgery and from the affected pelvis were significantly higher than nonoperated patients and controls. Urinary levels of OPN were significantly higher in surgical cases than in nonoperated patients. Urinary RANTES was significantly higher in samples from affected pelvis during surgery than in voided urine before pyeloplasty. Three months after surgery, no significant changes were detected

Ref.: reference number.

**Table 2:** Studies on urinary cytokines in patients with vesicoureteral reflux (VUR).

Author	Year	Ref.	Age of patients	Cytokine	Study/control group (*N*)	Sensitivity	Specificity	Conclusions
Haraoka et al.	1996	[84]	Mean age 6.7 years	IL-8	32/—	—	—	Levels of IL-8 are elevated in patients with VUR or renal scarring

Ninan et al.	1999	[85]	5 months to 13.33 years	IL-6; TNF-*α*	17/healthy children (15)	—	—	Levels of IL-6 and TNF-*α* receptor-1 are elevated in reflux associated with renal damage

Wang et al.	2001	[86]	Mean age 14.6 years	IL-6	66/healthy children (28)			Levels of IL-6 are elevated in severe bilateral renal scarring

Galanakis et al.	2006	[87]	1 month to 2 years	IL-8	24/ITU+/VUR− (14); ITU−/VUR− (21)	88%	69%	Levels of IL-8 are elevated in VUR patients

Sabasiñska et al.	2008	[88]	6.23 ± 4.15 years	TGF-*β*_1_	54/healthy children (27)	—	—	Highest urinary concentrations of TGF-*β*_1_ are detected in grades IV and V reflux

Gokce et al.	2010	[89]	1 month 16 years	IL-6; IL-8	87/healthy children (27)	—	—	IL-6 levels are elevated in VUR and IL-8 levels in renal scarring

Merrikhi et al.	2012	[90]	ITU+/RVU+: 4.3 ± 2.9; ITU+/RVU−: 4 ± 2.6; control group: 4 ± 2.1	IL-8	28 (ITU+/VUR+); 28 (ITU+/VUR−); 28 healthy	71.4% (cutoff point: 3 pg/*µ*moL)	58.9% (cutoff point: 3 pg/*µ*moL)	IL-8 levels were significantly higher in patients with RVU. At the cutoff point of 3 pg/*µ*moL, IL-8 was accurate in detecting VUR

Tramma et al.	2012	[91]	71 ± 42.5 months	IL-6; IL-8	50/history of pyelonephritis (23 RS+/VUR+; 10 RS+/VUR−; 13 RS−/VUR−; 4RS−/VUR+)	—	—	Urinary levels of IL-8 were undetectable in all samples. There were no differences between urinary IL-6 levels in children with or without VUR. The levels of IL-6 were directly correlated with the grade of renal scars

Ref.: reference number.

## References

[1] Estrada CR (2008). Prenatal hydronephrosis: early evaluation. *Current Opinion in Urology*.

[2] Lee RS, Cendron M, Kinnamon DD, Nguyen HT (2006). Antenatal hydronephrosis as a predictor of postnatal outcome: a meta-analysis. *Pediatrics*.

[3] Mallik M, Watson AR (2008). Antenatally detected urinary tract abnormalities: more detection but less action. *Pediatric Nephrology*.

[4] Cohen-Overbeek TE, Wijngaard-Boom P, Ursem NTC, Hop WCJ, Wladimiroff JW, Wolffenbuttel KP (2005). Mild renal pyelectasis in the second trimester: determination of cut-off levels for postnatal referral. *Ultrasound in Obstetrics and Gynecology*.

[5] Bouzada MCF, Oliveira EA, Pereira AK (2004). Diagnostic accuracy of fetal renal pelvis anteroposterior diameter as a predictor of uropathy: a prospective study. *Ultrasound in Obstetrics and Gynecology*.

[6] Coplen DE, Austin PF, Yan Y, Blanco VM, Dicke JM (2006). The magnitude of fetal renal pelvic dilatation can identify obstructive postnatal hydronephrosis, and direct postnatal evaluation and management. *Journal of Urology*.

[7] Gramellini D, Fieni S, Caforio E (2006). Diagnostic accuracy of fetal renal pelvis anteroposterior diameter as a predictor of significant postnatal nephrouropathy: second versus third trimester of pregnancy. *The American Journal of Obstetrics and Gynecology*.

[8] Nguyen HT, Herndon CDA, Cooper C (2010). The society for fetal urology consensus statement on the evaluation and management of antenatal hydronephrosis. *Journal of Pediatric Urology*.

[9] Dias CS, Silva JMP, Pereira AK (2013). Diagnostic accuracy of renal pelvic dilatation for detecting surgically managed ureteropelvic junction obstruction. *Journal of Urology*.

[10] Dias CS, Bouzada MCF, Pereira AK (2009). Predictive factors for vesicoureteral reflux and prenatally diagnosed renal pelvic dilatation. *Journal of Urology*.

[11] Phan V, Traubici J, Hershenfield B, Stephens D, Rosenblum ND, Geary DF (2003). Vesicoureteral reflux in infants with isolated antenatal hydronephrosis. *Pediatric Nephrology*.

[12] Coelho GM, Bouzada MCF, Lemos GS, Pereira AK, Lima BP, Oliveira EA (2008). Risk factors for urinary tract infection in children with prenatal renal pelvic dilatation. *Journal of Urology*.

[13] Farhat W, McLorie G, Geary D (2000). The natural history of neonatal vesicoureteral reflux associated with antenatal hydronephrosis. *Journal of Urology*.

[14] Ismaili K, Hall M, Donner C, Thomas D, Vermeylen D, Avni FE (2003). Results of systematic screening for minor degrees of fetal renal pelvis dilatation in an unselected population. *The American Journal of Obstetrics and Gynecology*.

[15] Moorthy I, Joshi N, Cook JV, Warren M (2003). Antenatal hydronephrosis: negative predictive value of normal postnatal ultrasound—a 5-year study. *Clinical Radiology*.

[16] Tibballs JM, de Bruyn R (1996). Primary vesicoureteric reflux—how useful is postnatal ultrasound?. *Archives of Disease in Childhood*.

[17] Toiviainen-Salo S, Garel L, Grignon A (2004). Fetal hydronephrosis: is there hope for consensus?. *Pediatric Radiology*.

[18] Leung VY, Chu WC, Metreweli C (2009). Hydronephrosis index: a better physiological reference in antenatal ultrasound for assessment of fetal hydronephrosis. *Journal of Pediatrics*.

[19] Ismaili K, Hall M, Piepsz A, Alexander M, Schulman C, Avni FE (2005). Insights into the pathogenesis and natural history of fetuses with renal pelvis dilatation. *European Urology*.

[20] Hubert KC, Palmer JS (2007). Current diagnosis and management of fetal genitourinary abnormalities. *Urologic Clinics of North America*.

[21] Piepsz A (2007). Antenatally detected hydronephrosis. *Seminars in Nuclear Medicine*.

[22] Lee RS (2009). Biomarkers for pediatric urological disease. *Current Opinion in Urology*.

[23] LaBaer J (2005). So, you want to look for biomarkers (introduction to the special biomarkers issue). *Journal of Proteome Research*.

[24] Muller F, Dreux S, Audibert F (2004). Fetal serum *β*2-microglobulin and cystatin C in the prediction of post-natal renal function in bilateral hypoplasia and hyperechogenic enlarged kidneys. *Prenatal Diagnosis*.

[25] Mesrobian HGO, Mitchell ME, See WA (2010). Candidate urinary biomarker discovery in ureteropelvic junction obstruction: a proteomic approach. *Journal of Urology*.

[26] Almodhen F, Loutochin O, Capolicchio JP, Jednak R, El-Sherbiny M (2009). The role of bladder urine transforming growth factor-*β*1 concentrations in diagnosis and management of unilateral prenatal hydronephrosis. *Journal of Urology*.

[27] Wen JG, Frøkiær J, Jørgensen TM, Djurhuus JC (1999). Obstructive nephropathy: an update of the experimental research. *Urological Research*.

[28] Klahr S, Morrissey J (2002). Obstructive nephropathy and renal fibrosis. *The American Journal of Physiology—Renal Physiology*.

[29] Alberti C (2012). Congenital ureteropelvic junction obstruction: physiopathology, decoupling of tout court pelvic dilatation-obstruction semantic connection, biomarkers to predict renal damage evolution. *European Review for Medical and Pharmacological Sciences*.

[30] Chevalier RL, Thornhill BA, Forbes MS, Kiley SC (2010). Mechanisms of renal injury and progression of renal disease in congenital obstructive nephropathy. *Pediatric Nephrology*.

[31] Matsell DG, Tarantal AF (2002). Experimental models of fetal obstructive nephropathy. *Pediatric Nephrology*.

[32] Klahr S (1998). Obstructive nephropathy. *Kidney International*.

[33] Borish LC, Steinke JW (2003). Cytokines and chemokines. *Journal of Allergy and Clinical Immunology*.

[34] Segerer S, Alpers CE (2003). Chemokines and chemokine receptors in renal pathology. *Current Opinion in Nephrology and Hypertension*.

[35] Segerer S, Nelson PJ, Schlöndorff D (2000). Chemokines, chemokine receptors, and renal disease: from basic science to pathophysiologic and therapeutic studies. *Journal of the American Society of Nephrology*.

[36] Viedt C, Dechend R, Fei J, Hänsch GM, Kreuzer J, Orth SR (2002). MCP-1 induces inflammatory activation of human tubular epithelial cells: involvement of the transcription factors, nuclear factor-*κ*B and activating protein-1. *Journal of the American Society of Nephrology*.

[37] Gröne HJ, Cohen CD, Gröne E (2002). Spatial and temporally restricted expression of chemokines and chemokine receptors in the developing human kidney. *Journal of the American Society of Nephrology*.

[38] Sheu JN, Chen MC, Cheng SL, Lee IC, Chen SM, Tsay GJ (2007). Urine interleukin-1*β* in children with acute pyelonephritis and renal scarring. *Nephrology*.

[39] Sheu JN, Chen MC, Lue KH (2006). Serum and urine levels of interleukin-6 and interleukin-8 in children with acute pyelonephritis. *Cytokine*.

[40] Souto MFO, Teixeira AL, Russo RC (2008). Immune mediators in idiopathic nephrotic syndrome: evidence for a relation between interleukin 8 and proteinuria. *Pediatric Research*.

[41] Decramer S, Bascands JI, Schanstra JP (2007). Non-invasive markers of ureteropelvic junction obstruction. *World Journal of Urology*.

[42] Artifoni L, Negrisolo S, Montini G (2007). Interleukin-8 and CXCR1 receptor functional polymorphisms and susceptibility to acute pyelonephritis. *Journal of Urology*.

[43] Monga M, Gabal-Shehab LL, Stein P (2001). Urinary transforming growth factor-*β*1 levels correlate with bladder outlet obstruction. *International Journal of Urology*.

[44] Klahr S, Morrissey JJ (1997). Comparative study of ACE inhibitors and angiotensin II receptor antagonists in interstitial scarring. *Kidney International, Supplement*.

[45] Miyajima A, Chen J, Lawrence C (2000). Antibody to transforming growth factor-*β* ameliorates tubular apoptosis in unilateral ureteral obstruction. *Kidney International*.

[46] Trachtman H, Weiser AC, Valderrama E, Morgado M, Palmer LS (2004). Prevention of renal fibrosis by spironolactone in mice with complete unilateral ureteral obstruction. *Journal of Urology*.

[47] Seseke F, Thelen P, Heuser M, Zöller G, Ringert RH (2001). Impaired nephrogenesis in rats with congenital obstructive uropathy. *Journal of Urology*.

[48] Chevalier RL, Thornhill BA, Chang AY, Cachat F, Lackey A (2002). Recovery from release of ureteral obstruction in the rat: relationship to nephrogenesis. *Kidney International*.

[49] Stephan M, Conrad S, Eggert T, Heuer R, Fernandez S, Huland H (2002). Urinary concentration and tissue messenger RNA expression of monocyte chemoattractant protein-1 as an indicator of the degree of hydronephrotic atrophy in partial ureteral obstruction. *Journal of Urology*.

[50] Seseke F, Thelen P, Hemmerlein B, Kliese D, Zöller G, Ringert RH (2000). Histologic and molecular evidence of obstructive uropathy in rats with hereditary congenital hydronephrosis. *Urological Research*.

[51] Chevalier RL, Thornhill BA, Wolstenholme JT, Kim A (1999). Unilateral ureteral obstruction in early development alters renal growth: dependence on the duration of obstruction. *Journal of Urology*.

[52] Zhou Y, Takahashi G, Shinagawa T (2002). Increased transforming growth factor-*β*1 and tubulointerstitial fibrosis in rats with congenital hydronephrosis. *International Journal of Urology*.

[53] Truong LD, Petrusevska G, Yang G (1996). Cell apoptosis and proliferation in experimental chronic obstructive uropathy. *Kidney International*.

[54] Truong LD, Choi YJ, Chun CC (2001). Renal cell apoptosis in chronic obstructive uropathy: the roles of caspases. *Kidney International*.

[55] Chevalier RL, Goyal S, Wolstenholme JT, Thornhill BA (1998). Obstructive nephropathy in the neonatal rat is attenuated by epidermal growth factor. *Kidney International*.

[56] Seremetis GM, Maizels M (1996). TGF-*β* mRNA expression in the renal pelvis after experimental and clinical ureteropelvic junction obstruction. *Journal of Urology*.

[57] Isaka Y, Tsujie M, Ando Y (2000). Transforming growth factor-*β*1 antisense oligodeoxynucleotides block interstitial fibrosis in unilateral ureteral obstruction. *Kidney International*.

[58] Mizuno S, Matsumoto K, Nakamura T (2001). Hepatocyte growth factor suppresses interstitial fibrosis in a mouse model of obstructive nephropathy. *Kidney International*.

[59] Derynck R, Zhang Y, Feng XH (1998). Smads: transcriptional activators of TGF-*β* responses. *Cell*.

[60] Sato M, Muragaki Y, Saika S, Roberts AB, Ooshima A (2003). Targeted disruption of TGF-*β*1/Smad3 signaling protects against renal tubulointerstitial fibrosis induced by unilateral ureteral obstruction. *Journal of Clinical Investigation*.

[61] Misseri R, Meldrum DR, Dagher P, Hile K, Rink RC, Meldrum KK (2004). Unilateral ureteral obstruction induces renal tubular cell production of tumor necrosis factor-*α* independent of inflammatory cell infiltration. *Journal of Urology*.

[62] Vielhauer V, Anders HJ, Mack M (2001). Obstructive nephropathy in the mouse: progressive fibrosis correlates with tubulointerstitial chemokine expression and accumulation of CC chemokine receptor 2- and 5-positive leukocytes. *Journal of the American Society of Nephrology*.

[63] Crisman JM, Richards LL, Valach DP, Franzoni DF, Diamond JR (2001). Chemokine expression in the obstructed kidney. *Experimental Nephrology*.

[64] Lauder AJ, Jolin HE, Smith P (2004). Lymphomagenesis, hydronephrosis, and autoantibodies result from dysregulation of IL-9 and are differentially dependent on Th2 cytokines. *Journal of Immunology*.

[65] Madsen MG (2013). Urinary biomarkers in hydronephrosis. *Danish Medical Journal*.

[66] Chuang YH, Chuang WL, Huang SP, Liu CK, Huang CH (2011). Atorvastatin ameliorates tissue damage of obstructed ureter in rats. *Life Sciences*.

[67] Hochberg D, Johnson CW, Chen J (2000). Interstitial fibrosis of unilateral ureteral obstruction is exacerbated in kidneys of mice lacking the gene for inducible nitric oxide synthase. *Laboratory Investigation*.

[68] Bulas DI, Fonda JS (1997). Prenatal evaluation of fetal anomalies. *Pediatric Clinics of North America*.

[69] Zhang PL, Peters CA, Rosen S (2000). Ureteropelvic junction obstruction: morphological and clinical studies. *Pediatric Nephrology*.

[70] Grandaliano G, Gesualdo L, Bartoli F (2000). MCP-1 and EGF renal expression and urine excretion in human congenital obstructive nephropathy. *Kidney International*.

[71] Bartoli F, Gesualdo L, Paradies G (2000). Renal expression of monocyte chemotactic protein-1 and epidermal growth factor in children with obstructive hydronephrosis. *Journal of Pediatric Surgery*.

[72] Bartoli F, Penza R, Aceto G (2011). Urinary epidermal growth factor, monocyte chemotactic protein-1, and *β*2-microglobulin in children with ureteropelvic junction obstruction. *Journal of Pediatric Surgery*.

[73] Madsen MG, Nørregaard R, Palmfeldt J, Olsen LH, Frøkiær J, Jørgensen TM (2012). Epidermal growth factor and monocyte chemotactic peptide-1: potential biomarkers of urinary tract obstruction in children with hydronephrosis. *Journal of Pediatric Urology*.

[74] Taranta-Janusz K, Wasilewska A, Dębek W, Waszkiewicz-Stojda M (2012). Urinary cytokine profiles in unilateral congenital hydronephrosis. *Pediatric Nephrology*.

[75] Palmer LS, Maizels M, Kaplan WE, Firlit CF, Cheng EY (1997). Urine levels of transforming growth factor-*β* 1 in children with ureteropelvic junction obstruction. *Urology*.

[76] Furness PD, Maizels M, Han SW, Cohn RA, Cheng EY (1999). Elevated bladder urine concentration of transforming growth factor-*β*1 correlates with upper urinary tract obstruction in children. *Journal of Urology*.

[77] Sager C, Lopez JC, Duran V, Burek C, Perazzo E (2009). Transforming growth factor-*β*1 in congenital ureteropelvic junction obstruction: diagnosis and follow-up. *International Brazilian Journal of Urology*.

[78] El-Sherbiny MT, Mousa OM, Shokeir AA, Ghoneim MA (2002). Role of urinary transforming growth factor-*β*1 concentration in the diagnosis of upper urinary tract obstruction in children. *Journal of Urology*.

[79] Taha MA, Shokeir AA, Osman HG, Abd El-Aziz AEF, Farahat SE (2007). Pelvi-ureteric junction obstruction in children: the role of urinary transforming growth factor-*β*1 and epidermal growth factor. *BJU International*.

[80] Zieg J, Blahova K, Seeman T (2011). Urinary transforming growth factor-*β*1 in children with obstructive uropathy. *Nephrology*.

[81] Elder JS, Peters CA, Arant BS (1997). Pediatric vesicoureteral reflux guidelines panel summary report on the management of primary vesicoureteral reflux in children. *Journal of Urology*.

[82] Silva JMP, Diniz JSS, Simões e Silva AC, Azevedo MV, Pimenta MR, Oliveira EA (2006). Predictive factors of chronic kidney disease in severe vesicoureteral reflux. *Pediatric Nephrology*.

[83] Chertin B, Farkas A, Puri P (2003). Epidermal growth factor and monocyte chemotactic peptide-1 expression in reflux nephropathy. *European Urology*.

[84] Haraoka M, Senoh K, Ogata N, Furukawa M, Matsumoto T, Kumazawa J (1996). Elevated interleukin-8 levels in the urine of children with renal scarring and/or vesicoureteral reflux. *Journal of Urology*.

[85] Ninan GK, Jutley RS, Eremin O (1999). Urinary cytokines as markers of reflux nephropathy. *Journal of Urology*.

[86] Wang J, Konda R, Sato H, Sakai K, Ito S, Orikasa S (2001). Clinical significance of urinary interleukin-6 in children with reflux nephropathy. *Journal of Urology*.

[87] Galanakis E, Bitsori M, Dimitriou H, Giannakopoulou C, Karkavitsas NS, Kalmanti M (2006). Urine interleukin-8 as a marker of vesicoureteral reflux in infants. *Pediatrics*.

[88] Sabasiñska A, Zoch-Zwierz W, Wasilewska A, Porowski T (2008). Laminin and transforming growth factor *β*-1 in children with vesicoureteric reflux. *Pediatric Nephrology*.

[89] Gokce I, Alpay H, Biyikli N, Unluguzel G, Dede F, Topuzoglu A (2010). Urinary levels of interleukin-6 and interleukin-8 in patients with vesicoureteral reflux and renal parenchymal scar. *Pediatric Nephrology*.

[90] Merrikhi AR, Keivanfar M, Gheissari A, Mousavinasab F (2012). Urine interlukein-8 as a diagnostic test for vesicoureteral reflux in children. *Journal of the Pakistan Medical Association*.

[91] Tramma D, Hatzistylianou M, Gerasimou G, Lafazanis V (2012). Interleukin-6 and interleukin-8 levels in the urine of children with renal scarring. *Pediatric Nephrology*.

[92] Yim HE, Bae IS, Yoo KH, Hong YS, Lee JW (2007). Genetic control of VEGF and TGF-*β*1 gene polymorphisms in childhood urinary tract infection and vesicoureteral reflux. *Pediatric Research*.

[93] Kuroda S, Solari V, Puri P (2007). Association of transforming growth factor-*β*1 gene polymorphism with familial vesicoureteral reflux. *Journal of Urology*.

[94] Lee-Chen GJ, Liu PK, Lai YC, Juang HS, Huang SY, Lin CY (2004). Significance of the tissue kallikrein promoter and transforming growth factor-*β*1 polymorphisms with renal progression in children with vesicoureteral reflux. *Kidney International*.

[95] Solari V, Ennis S, Cascio S, Puri P (2004). Tumor necrosis factor-*α* gene polymorphism in reflux nephropathy. *Journal of Urology*.

[96] Hussein A, Askar E, Elsaeid M, Schaefer F (2010). Functional polymorphisms in transforming growth factor-*β*-1 (TGF*β*-1) and vascular endothelial growth factor (VEGF) genes modify risk of renal parenchymal scarring following childhood urinary tract infection. *Nephrology Dialysis Transplantation*.

[97] Schwentner C, Oswald J, Lunacek A (2008). Extracellular microenvironment and cytokine profile of the ureterovesical junction in children with vesicoureteral reflux. *Journal of Urology*.

[98] Vianna HR, Soares CM, Silveira KD (2013). Cytokines in chronic kidney disease: potential link of MCP-1 and dyslipidemia in glomerular diseases. *Pediatric Nephrology*.

[99] Jutley RS, Youngson GG, Eremin O, Ninan GK (2000). Serum cytokine profile in reflux nephropathy. *Pediatric Surgery International*.

[100] Vasconcelos MA, Bouzada MCF, Silveira KD (2011). Urinary levels of TGF *β*-1 and of cytokines in patients with prenatally detected nephrouropathies. *Pediatric Nephrology*.

[101] Harambat J, van Stralen KJ, Kim JJ, Tizard EJ (2012). Epidemiology of chronic kidney disease in children. *Pediatric Nephrology*.

[102] Daïkha-Dahmane F, Dommergues M, Muller F (1997). Development of human fetal kidney in obstructive uropathy: correlations with ultrasonography and urine biochemistry. *Kidney International*.

